# Supplementary material for: Computational design of pH-sensitive binders
Source: bioRxiv. 2025 Sep 29:2025.09.29.678932. Preprint. [Version 1] doi: 10.1101/2025.09.29.678932 (PMC12621740; doi:10.1101/2025.09.29.678932)
Supplement: Supplement 1 [file media-1.pdf]

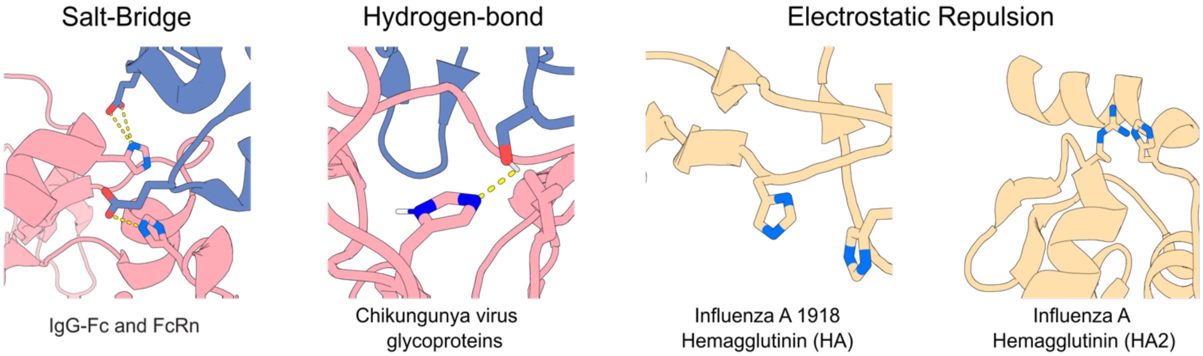

Fig S1. pH-dependent interactions found in native proteins. Salt-bridge is indicated in IgG-Fc and FcRn interaction[5] (PDB 1I1A). Hydrogen-bond network is shown in Chikungunya virus glycoproteins[6] (PDB 3N43). Electrostatic repulsion between histidine-histidine or histidine-arginine is shown for influenza A hemagglutinin complexes[7,8] (PDB 4FNK, 1RUY).

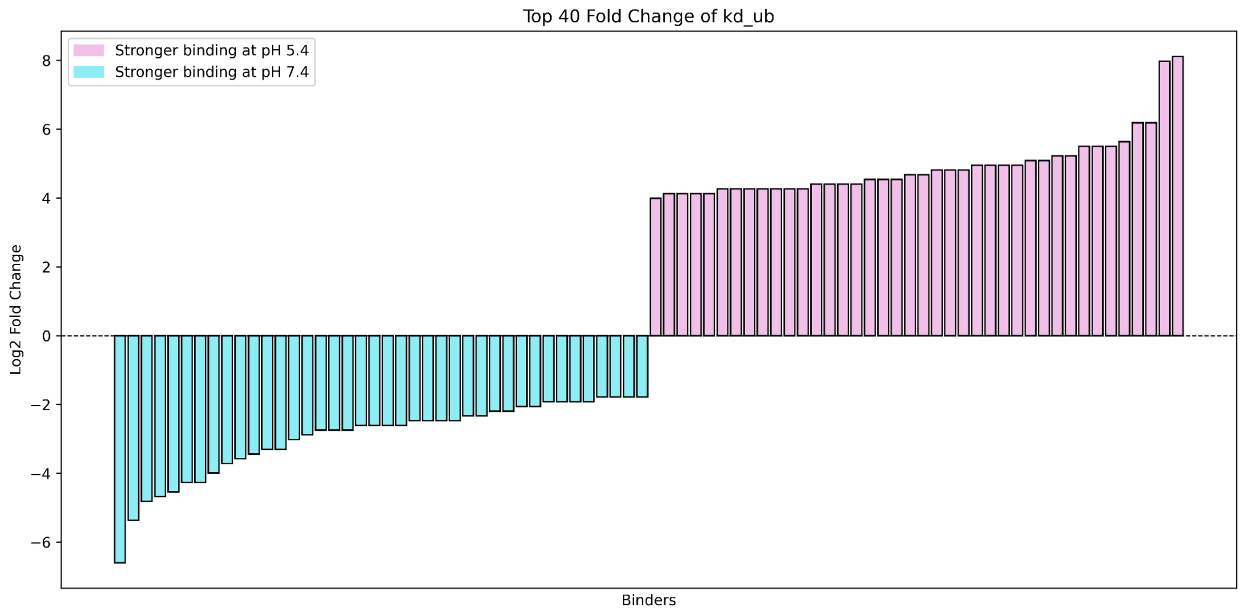

Fig. S2. **Yeast log fold change at pH 7.4 vs 5.4 for designed EphA2 binders.**

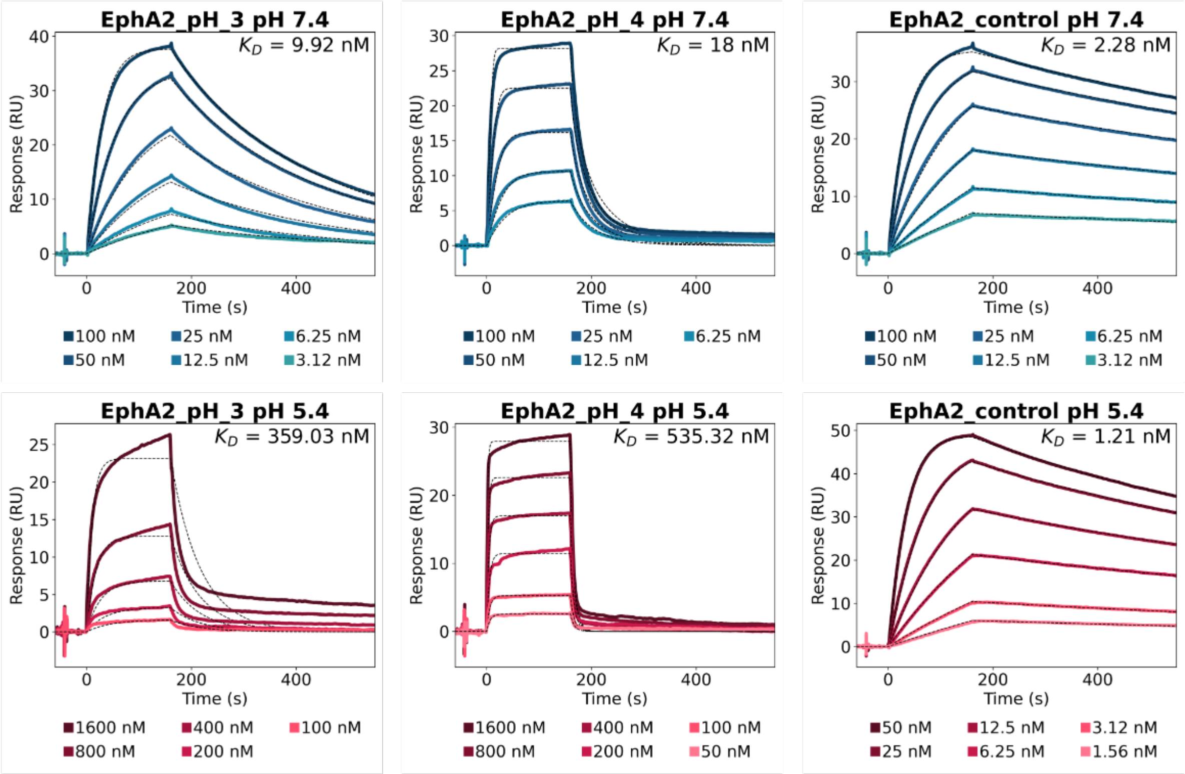

Fig S3. pH sensitive EphA2 binders. Binding affinities of designed pH-sensitive EphA2 binders_3, 4, control were determined by SPR at pH 7.4 and 5.4. EphA2_control was a pH insensitive binder utilized in future cell experiments.

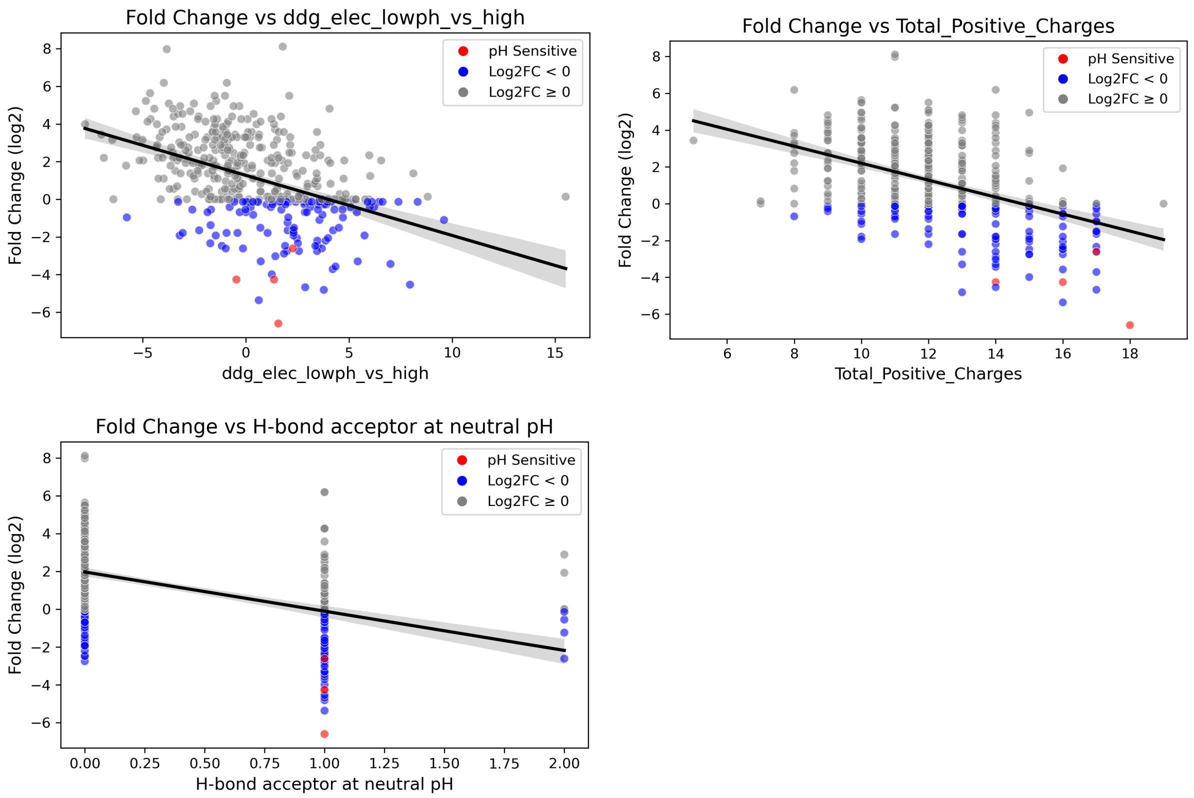

Fig S4. Metrics correlating with pH sensitivity. ddg_elec, positive charges, and H-bond acceptor at neutral pH were compared to the log2 fold change of binding from the EphA2 yeast library.

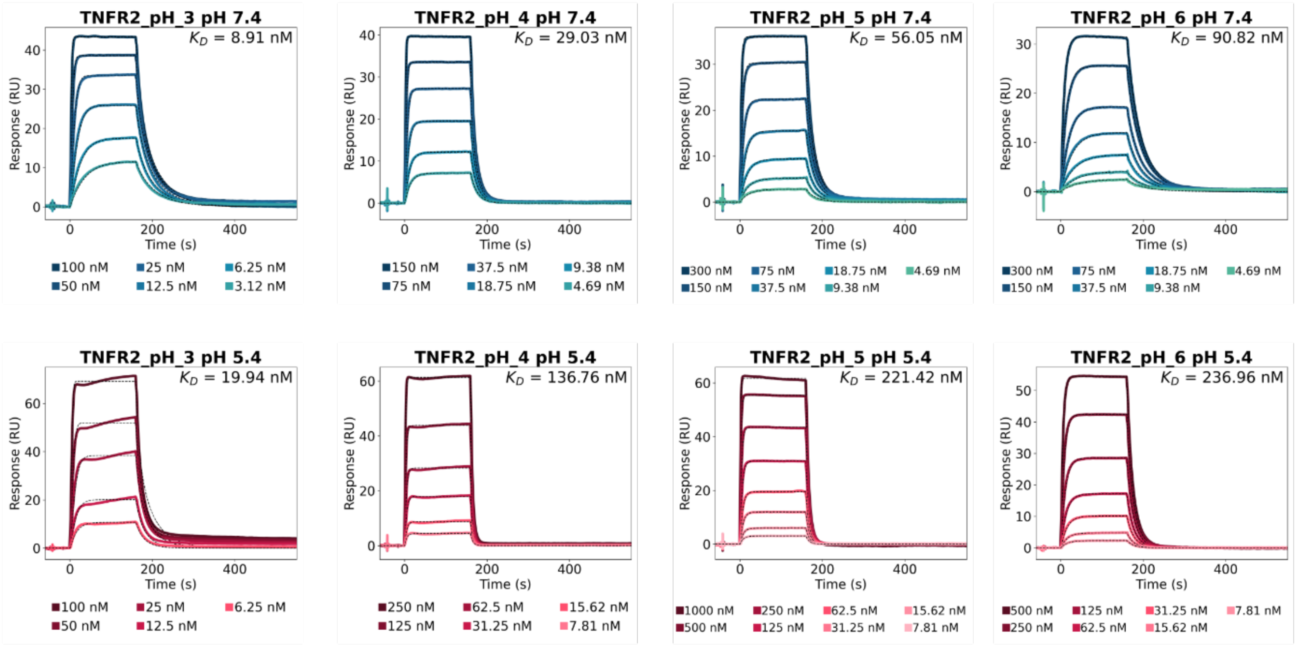

Fig S5. pH sensitive TNFR2 binders. Binding affinities of pH-sensitive TNFR2 binders_3, 4, 5, 6 were determined by SPR at pH 7.4 and 5.4.

A

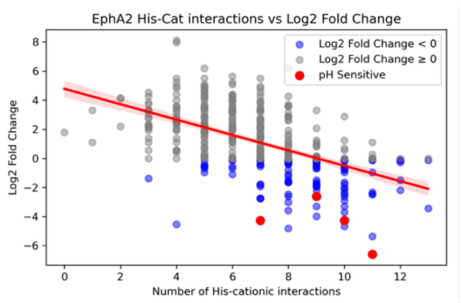

B

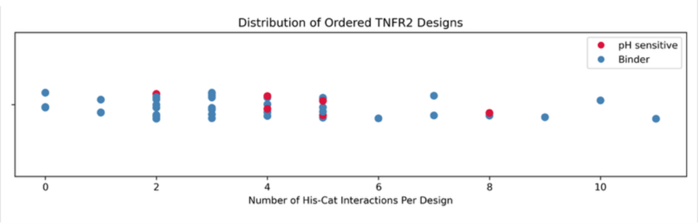

C

EphA2_pH_1 Binder Interactions

binder_residue	distance	target_residue
15H	3.7	142R
19H	7.8	199H
19H	7.6	198R
19H	8.6	201K
19H	8.8	142R
22H	8.7	199H
22H	4.9	198R

EphA2_pH_2 Binder Interactions

binder_residue	distance	target_residue
19H	7.6	199H
19H	5.3	198R
19H	8.2	201K
19H	8.9	142R
20H	8.8	198R
20H	9.8	142R

EphA2_pH_3 Binder Interactions

binder_residue	distance	target_residue
19H	7.6	199H
19H	7.3	198R
19H	8.4	201K
19H	8.9	142R
9H	9.1	142R

EphA2_pH_4 Binder Interactions

binder_residue	distance	target_residue
19H	7.7	199H
19H	7.0	198R
19H	8.3	201K
19H	8.9	142R

EphA2_control Binder Interactions

binder_residue	distance	target_residue
25H	5.7	115R
27H	10.0	105K
55H	5.9	116R

Fig S6. Calculated electrostatic (histidine-cationic) interactions across designed binders. **A.** Scatter plot comparing the log2 fold change of binding from the EphA2 yeast library with calculated his-cat interactions. pH-sensitive binders are colored in red. **B.** Distribution of ordered TNFR2 designs and the number of his-cat interactions. The SPR validated pH sensitive binders are colored in red. **C.** Distance in Å between indicated designed binder residue and target residue in 4 different pH-sensitive EphA2 binders and the pH-insensitive control binder. Distances were measured between non-carbon sidechain heavy atoms (HIS – ND1, NE2, ARG – NE, NH1, NH2, LYS – NZ).

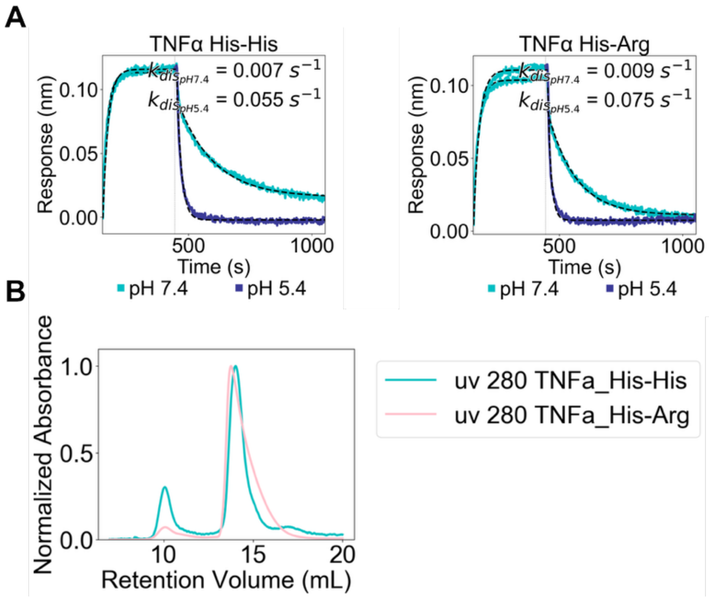

Fig S7. Characterizations of pH-sensitive TNFα binders. A. BLI curves of TNFα binders with His-His network or His-Arg network. Association at pH 7.4 and dissociation at 5.4 showed faster dissociation at pH 5.4. **B.** Size exclusion chromatography traces of pH-sensitive TNFα binders.

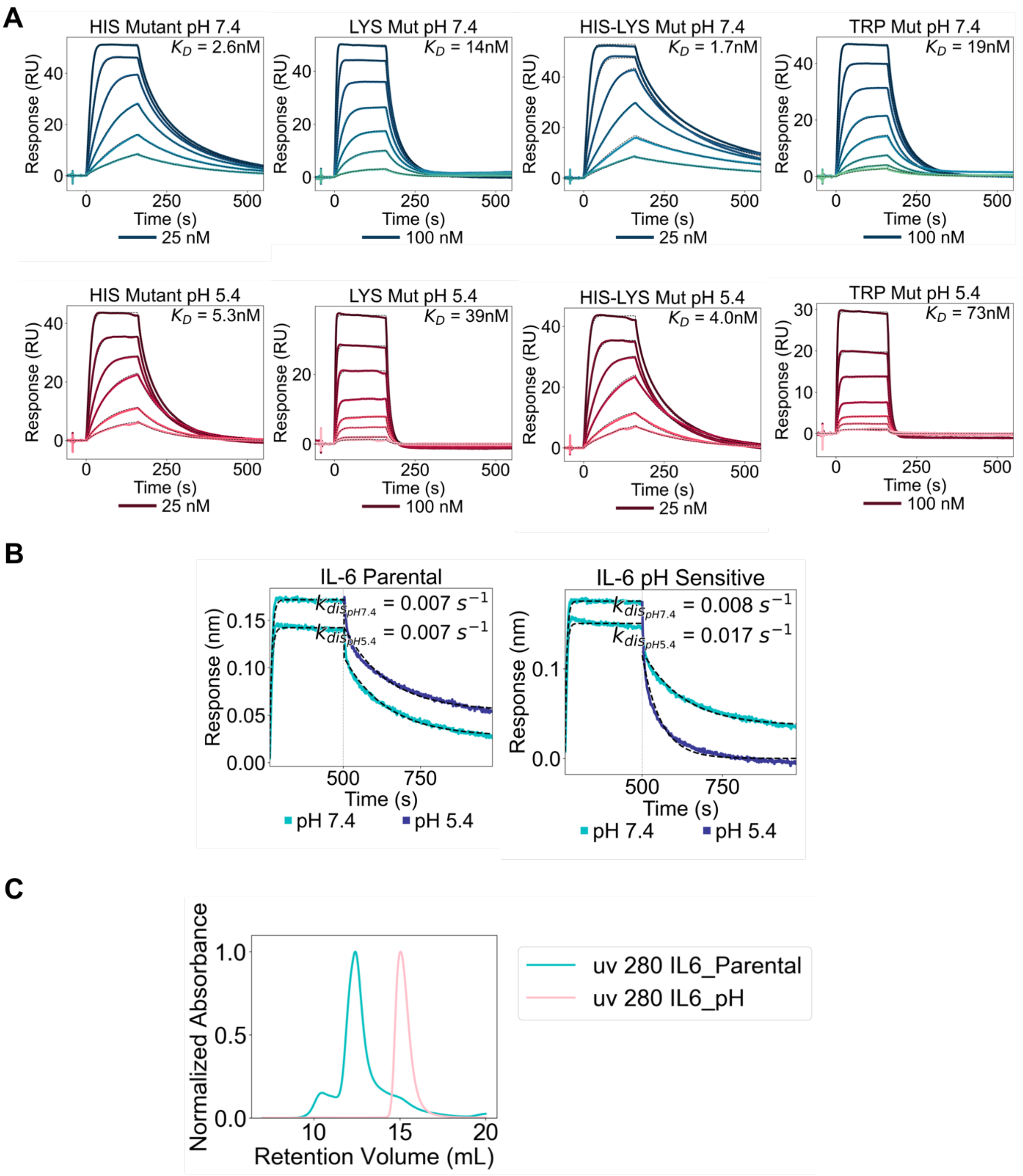

Fig S8. Characterizations of pH-sensitive IL-6 binders. A. SPR curves of pH sensitive IL-6 binder variants with the indicated residues restored to the parental sequence at pH 7.4 and 5.4. **B.** BLI curves of parental and pH-sensitive IL-6 binders. Association at pH 7.4 and dissociation at 5.4 showed faster dissociation at pH 5.4 for the pH sensitive binder. **C.** Size exclusion chromatography traces of the parental and pH-sensitive IL-6 binders.

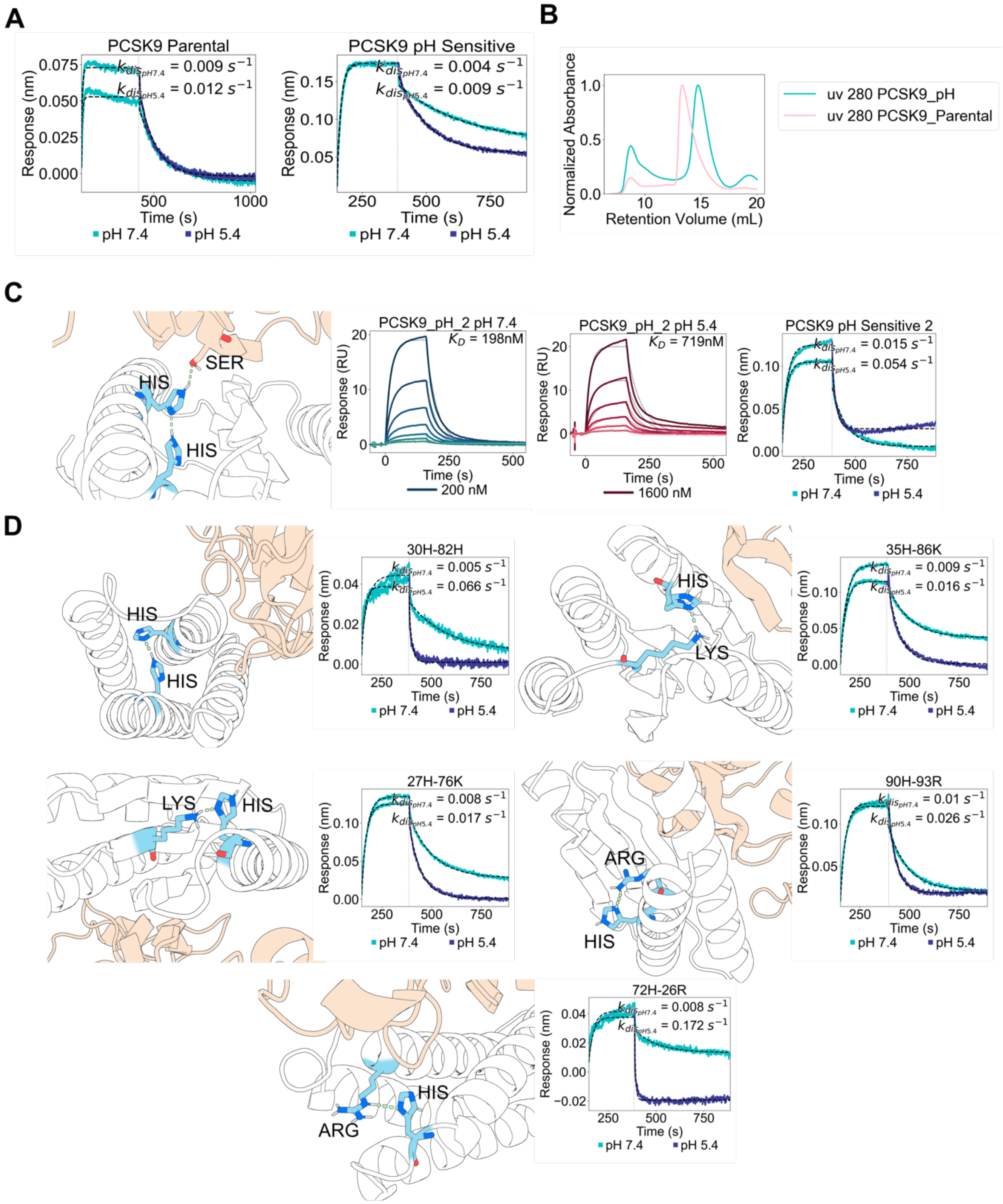

Fig. S9. Characterizations of pH-sensitive PCSK9 binders. A. BLI curves of parental and pH-sensitive PCSK9 binders. Association was conducted at pH 7.4 and dissociation at pH 5.4. **B.** Size exclusion chromatography traces of pH-sensitive PCSK9 binders. **C.** Design model and SPR curves of pH sensitive PCSK9 binder_2 at pH 7.4 and 5.4. BLI curves of association at pH 7.4 and dissociation at 5.4 showed faster dissociation at pH 5.4. **D.** Design model and BLI curves of pH-sensitive PCSK9 binders with various charged networks. Association at pH 7.4 and dissociation at 5.4 showed faster dissociation at pH 5.4.

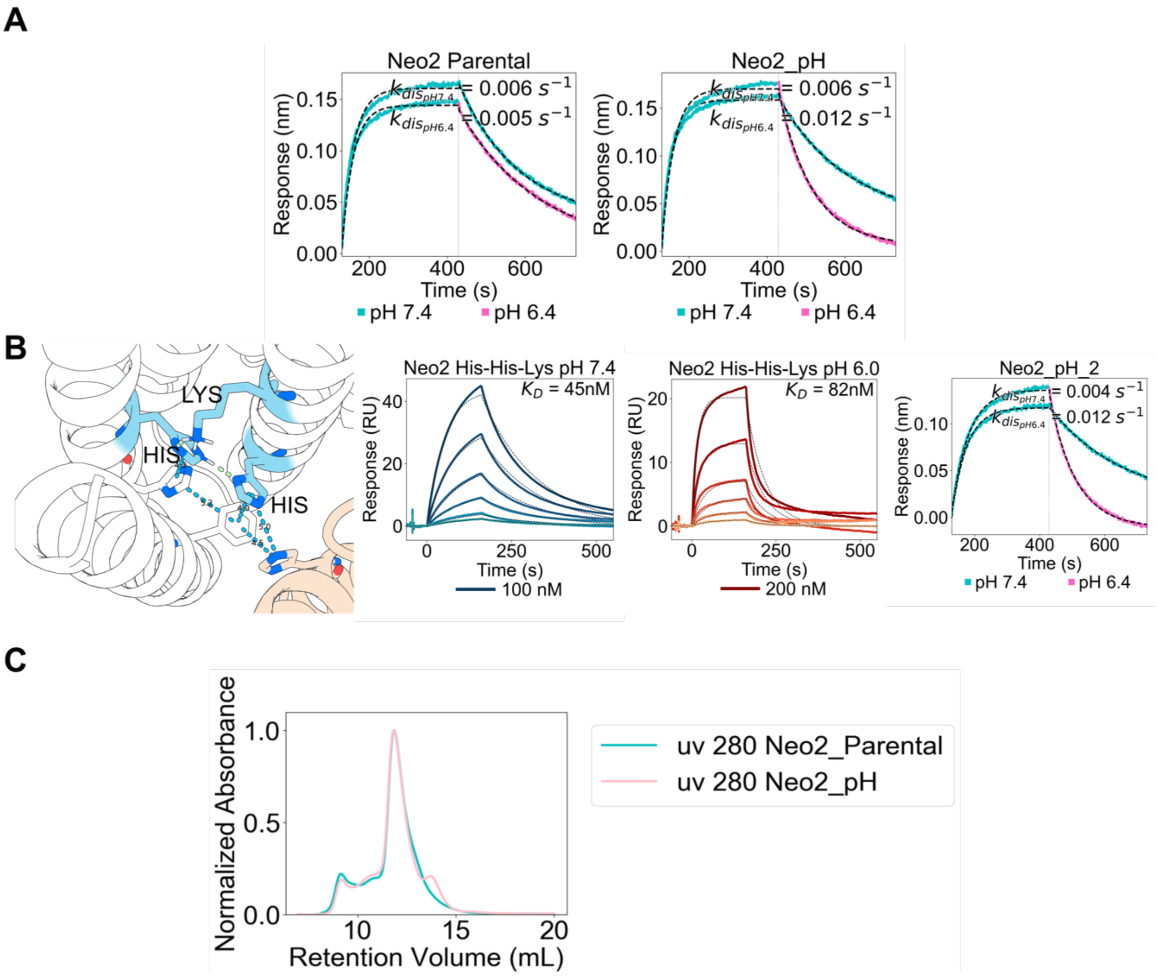

Fig. S10. Characterizations of pH-sensitive Neo2 binders. A. BLI curves of parental and pH-sensitive Neo2 binders. Association at pH 7.4 and dissociation at 6.4 showed faster dissociation at pH 6.4 for pH-sensitive binder. **B.** Design model and SPR curves of pH-sensitive Neo2 binder_2 with His-His-Lys network at pH 7.4 and 6.0. Association at pH 7.4 and dissociation at 6.4 showed faster dissociation at pH 6.4 for pH-sensitive binders. **C.** Size exclusion chromatography traces of pH-sensitive Neo2 binders.

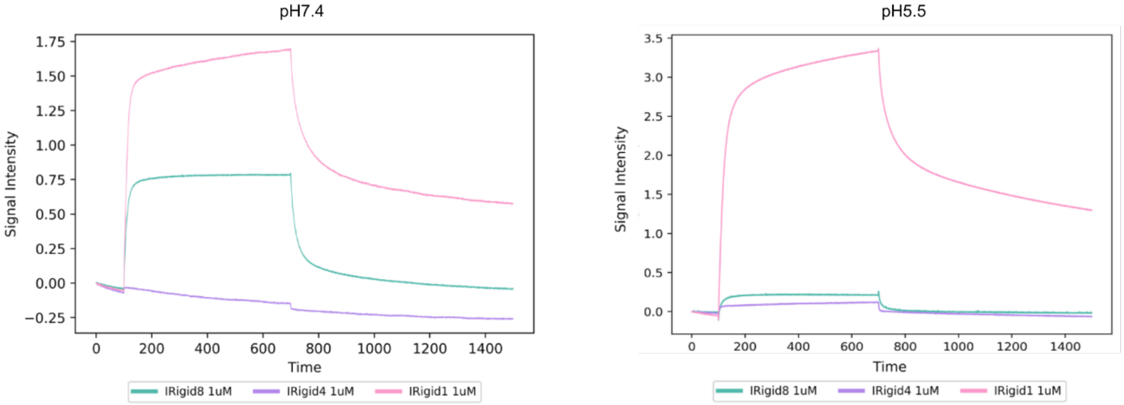

Fig. S11. BLI binding curve of IGF2R Rigid (IRigid) binders at pH 7.4 and pH 5.5 to domain 11 of IGF2R.

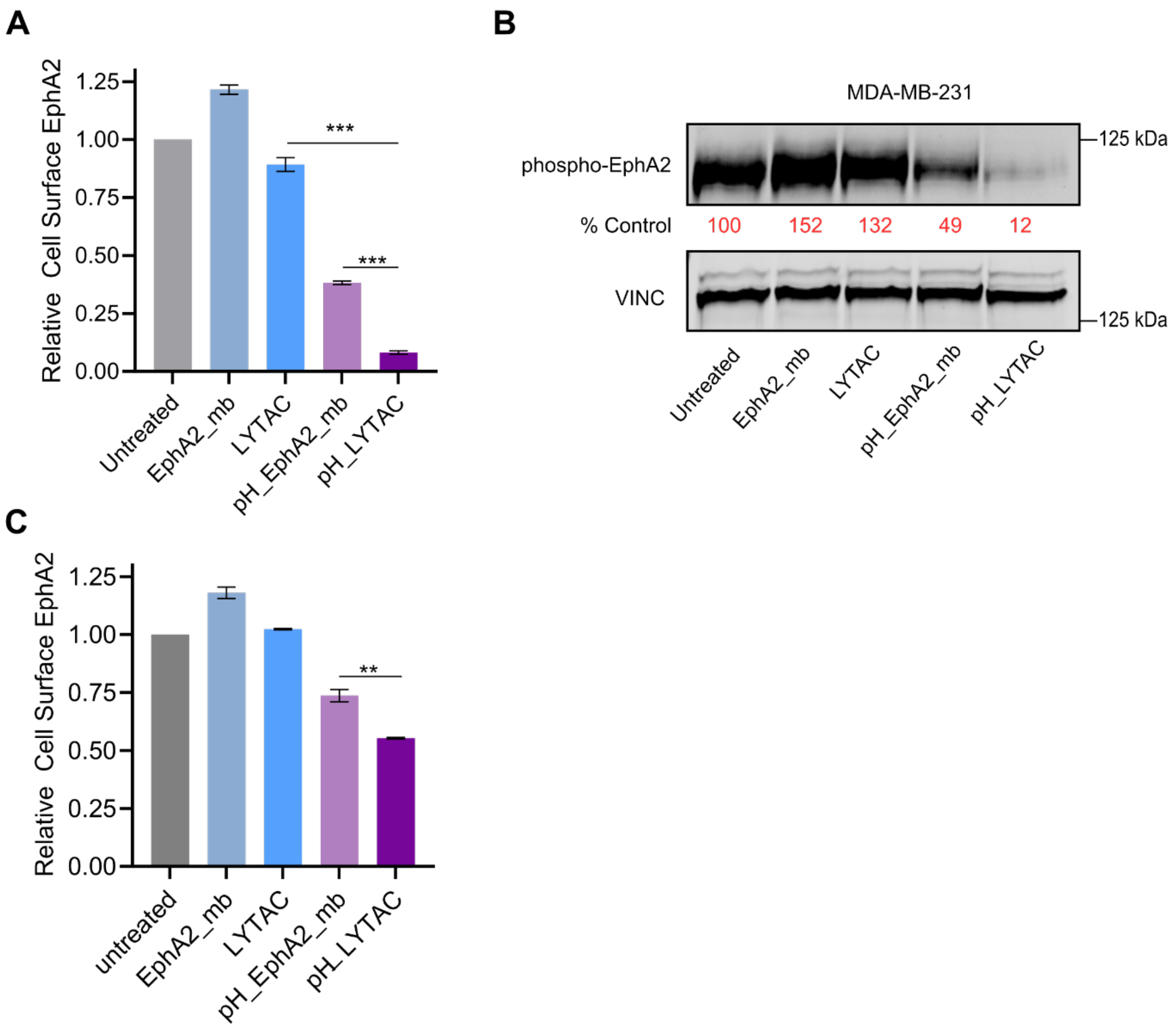

Fig S12. Degradation of EphA2 in MDA-MB-231 cells. A. Degradation of cell-surface EphA2 in MDA-MB-231 cells as determined by flow cytometry following 24h of 250 nM treatment. **B.** Degradation of phosphorylated EphA2 in MDA-MB-231 cells as determined by western blot following 24h of 250 nM treatment. **C.** Degradation of cell-surface EphA2 following a washout. MDA-MB-231 cells were treated with 250 nM of designed proteins for 24 h, then were washed out of extracellular treatment and incubated with fresh media for 6h.
